# Reframing dementia: Nursing students' relational learning with rather than about people with dementia. A constructivist grounded theory study

**DOI:** 10.1002/gps.5452

**Published:** 2020-11-11

**Authors:** Wendy Grosvenor, Ann Gallagher, Sube Banerjee

**Affiliations:** ^1^ School of Health Sciences Faculty of Health and Medical Sciences University of Surrey Guildford UK; ^2^ Faculty of Health University of Plymouth Plymouth UK

**Keywords:** Alzheimer's disease, dementia, healthcare education, long‐term conditions, nurse education, person‐centred care

## Abstract

**Objectives:**

Developing an informed and effective workforce that provides effective and ethical care to people with dementia and their families is an international priority. Here we explore the impact of a novel approach on students of adult nursing. It involved engagement with people with dementia and their carers over 3 years in the *Time for Dementia* Programme. This research explored students' perceptions of their professional learning and practice.

**Methods:**

A longitudinal, constructivist grounded theory approach in three phases (3 years) was used. In‐depth interviews were conducted with 12 students of adult nursing following visits with older adults with dementia and their carers in their own homes at 12 months, 24 months and at 36 months. A constant comparative analysis of transcribed interviews was completed.

**Results:**

A new theory of Whole Sight was identified as representing the impact of the learning that occurred as a consequence of relational learning visits. The core category of New Ways of Seeing dementia represented a broadening of students' views of dementia that encompassed the person's lives and relationships. This led to a person‐centred shift in students' practice. The data suggest that Time for Dementia can help students to be active in their contribution to care and serve as change agents in transforming dementia care.

**Conclusions:**

The theory of Whole Sight that emerged is a novel and useful contribution to the evidence on community‐based educational initiatives. Visiting people with dementia and their carers at home in training can help develop a workforce that meets their needs.

## 1 INTRODUCTION

Dementia is a global phenomenon affecting 46.8 million people worldwide and the care of people with dementia is a global concern.^1^, ^2^, ^3^, ^4^ The World Health Organisation^5^ has identified the development of the knowledge and skills of healthcare professionals in dementia as a priority. Collier et al.^6^ assert that high quality dementia education must underpin this ambition. Martin, O'Connor and Jackson's^4^ scoping review of gaps and priorities in dementia care in Europe, highlighted a *critical need to empower staff in dementia care,* arguing that lack of confidence is often associated with dehumanising care in practice. In the United Kingdom (UK), the *National Dementia Strategy for England* ^7^ identified deficiencies in dementia skills and knowledge by healthcare professionals. Subsequent analyses of education and training by the Department of Health and *Skills for Care* ^8^ highlighted that the most significant gap to address was the lack of dementia education at the early stages of student education. This view is supported by more recent reports that health and social care professionals, once qualified, do not feel adequately prepared to care for people with dementia.^9^, ^10^, ^11^

**Key points**

- Many health care professionals, once qualified, do not feel adequately prepared to care for people with dementia and highlights a gap in the lack of dementia education during student education
- This study explores the impact of learning with people with dementia and their carers as an educational intervention that challenges students' assumptions about dementia
- Results highlight that the best people to prepare future nurses are those living with dementia
- The impact of this model of dementia education in preparing future nurses to embed person‐centred care is highlighted and findings can inform future dementia education

Around the world, pre‐registration education programmes for nurse's anchor and set the standards for the future quality of healthcare provision by determining the knowledge, attitudes and competencies required to support people with dementia and their carers, and their value and priority.^2^ However, inconsistencies and gaps in professional dementia education have been highlighted internationally.^6^, ^12^, ^13^ Surr et al.^13^ identified that there appears to be little support or provision for regulation or quality monitoring of dementia education. There are unanswered questions about how to enable healthcare education to engage with the new and growing challenges posed by dementia. As Willis^14^ argues:

The education of our nursing and care workforce over the next ten years will determine the strength of our healthcare system for decades.

The absence of a cure for dementia increases the emphasis on quality of life as the overarching goal of dementia care practice. This study explores the effect of a novel educational approach, the *Time for Dementia* Programme that puts people with dementia and their family carers in the position of mentors and educators for healthcare students.^15^

### 1.1 Time for Dementia Programme

People with dementia and their carers were involved in the design of the programme from its conception and development through to its conduct and evaluation. In *Time for Dementia,* students visit a person with dementia and their family in pairs for 2 h every 3 months for 2 years, as well as receiving reflective practice sessions and supportive lectures.

Families are recruited from those accessing Alzheimer's Society support services and those in contact with National Health Service (NHS) mental health trusts in Kent, Surrey and Sussex and supported by the Alzheimer's Society. Paired visits were chosen to support joint learning and reflection. The programme was first established as a core component of the curricula for medical, nursing and paramedic students at two universities in the south of England. Students follow a visit guideline that includes focusing on life story work and effective and inclusive conversation with both the person with dementia and their carer. A detailed summary of the Time for Dementia Programme can be found in an earlier publication by Banerjee.^15^ NHS Health Research Authority Ethics approval was obtained (Ref 15/LO/0046).

Here we report the results of a qualitative study which aimed to generate an understanding of the impact on the knowledge and practice of students of adult nursing of the longitudinal visiting of people living with dementia and their carers that is the core of *Time for Dementia*.

## 2 METHOD

### 2.1 Design

**FIGURE 1:**
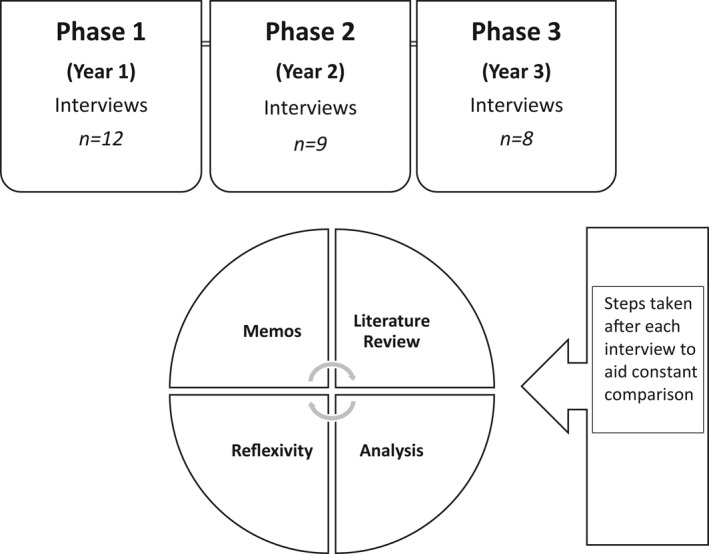
Summary of phases and data analysis

This was a longitudinal qualitative study with three data collection points that followed 12 undergraduate students of adult nursing. Data were collected during face‐to‐face interviews (*n* = 28). A constructivist grounded theory approach was adopted for this study to ensure that student nurse participants were key players in shaping the findings of the study.^16^ The study involved successive interviews with students visiting the same person and their carer over time and with the progression of the illness. The longitudinal phases and data analysis of the study are summarised in Figure 1.

### 2.2 Ethical considerations

The study was reviewed and granted ethical approval by the University Ethics Committee of a university in the south of England.

### 2.3 Sample and setting

Students of adult nursing were recruited from participants of the *Time for Dementia* programme at a university in the south of England. Charmaz^17^ suggests approximately 25 interviews is sufficient for a medium sized study, supported by Stern^18^ who advocates that 20–30 h of observations are often enough to reach saturation. As the study utilised multiple longitudinal interviews with the same participants over 3 years 12 participants were recruited.

### 2.4 Procedure

The study was conducted over 3 years with each phase of data collected over approximately 12‐month intervals. Interviews were carried out annually between November 2014 and July 2017 and lasted 60 min. All interviews were audio‐recorded, transcribed verbatim and checked for accuracy. Returning several times through follow up interviews at Phase 2 and 3 was congruent with a constructivist approach to try to capture participants' evolving experiences.^19^ ^,^ ^20^, ^21^, ^22^

### 2.5 Data analysis

Because grounded theory is not a linear process, collection of data, analysis and development of theories occurred simultaneously. Coding of data was undertaken as suggested by Charmaz^23^ ^,^ ^24^ in two phases: initial and focused coding. An inductive rather than deductive approach to collection of data and analysis was used to ensure that the experiences of the students remained central to the development of a new theory for understanding the impact of visits.

All transcripts were analysed using grounded theory techniques which identified patterns of meaning. The use of participants' own language, as advocated by Elliott and Jordan,^22^ during the early stages of analysis was used to assist the robustness of concepts that emerged. Transcripts were coded using constant comparison techniques, which involved reviewing coding and data between existing and new transcripts to: (i) identify new codes; (ii) check the use of codes for consistency and (iii) explore relationships between different codes. Core conceptual categories were developed through repeatedly moving back and forth between the data categories as suggested by Charmaz,^24^ to refine our understanding of emerging themes to produce a final conceptual model.

## 3 RESULTS

### 3.1 Participants and interviews

Twelve undergraduate students of adult nursing were approached for interview at 3 yearly time points, 28 interviews were completed. The impact of using sequential interviews with the same participants added depth and resonance to participants' stories, helping to produce a fuller narrative. The approach used meant that emerging categories in each subsequent phase could be followed up. A summary of participants' profiles can be found in Table 1.

**TABLE 1:** Participant profiles—Interviews (Phase 1–3)

Participant ref number^a^	Age of participant (years)	Gender	Prior dementia experience (Personal or professional)
**1**	**31**	**F**	**None**
**2**	**30**	**F**	**Worked in a dementia care home**
**3**	**23**	**F**	**‘Bit’ volunteered 1 day a week in dementia residential home**
**4**	**18**	**F**	**Some work experience—mainly alcohol related dementia**
**5**	**25**	**F**	**None**
**6**	**18**	**F**	**‘Bit’‐work with people with learning disabilities, some had dementia**
**7**	**21**	**F**	**Minimal worked in independent assisted living home**
**8**	**38**	**F** ^b^	**None**
**9**	**19**	**F** ^b^	**Personal—grandparent**
**10**	**49**	**F**	**None**
**11**	**24**	**M** ^b^	**None**
**12**	**29**	**M** ^b^	**Personal—grandparent**

^a^ Assigned to participants to assure anonymity.

^b^ indicates participants from non‐white background.

### 3.2 Introducing the theory of *Whole Sight* and its model

**FIGURE 2:**
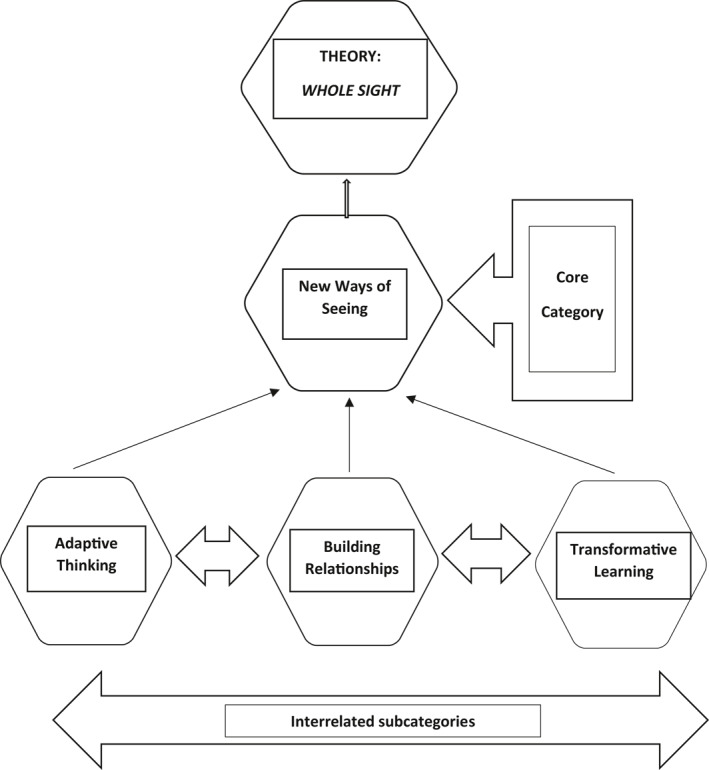
Impact on student nurses' learning and practice

The theory of *Whole Sight* was built around the core category of *New Ways of Seeing*, illustrating the impact of longitudinal visits on participants' professional learning and practice. As recommended by Glaser,^25^ to help improve understanding of the results, a graphical representation of the theory was developed to help communicate the results (Figure 2).

*Whole Sight* reflects how enabling participants to *step in their shoes* by entering the world of the person with dementia developed *moral imagination* as advocated by Murdock^26^:

Reflecting on how visits have impacted on me…there was a lady I was struggling with, I had to remember, she's someone's wife, mum…you have to remember they're human, you have to adapt to their world as they cannot adapt to ours. (P2, Phase 3)

Visits opened the homes of people with dementia as an aspect of the *real world,* which Schneebeli et al.^27^ suggests: *normalises the experience of illness, enhancing the development of the idea of patients as people*. These experiences resulted in participants' reframing their perceptions of dementia, as attention was given to broadening their views of dementia to encompass the person's lives and relationships, seeing and treating people with dementia as *wholes,* and not just focusing on their dementia.

See visits as helping to aid me develop as a caring nurse…importance of seeing and working with the whole person…**whole sight** I suppose. (P7, Phase 2)

### 3.3 Core category: *New Ways of Seeing*

As participants faced their assumptions of dementia, findings suggest this resulted in greater participant self‐awareness of the importance of *seeing the person* rather than their dementia. Participants frequently expressed how they were inspired to see beyond the persons' dementia, as a result of listening to the person, their perceptions of living with dementia became more individualised. The core category of *New Ways of Seeing,* illustrates the impact of entering the world of a person with dementia.

(I) see dementia in a different light […] realise that we often disable people with dementia in practice, assumption is they cannot perform own care tasks. Visits have helped me to reframe dementia […] focus on the person more and what is important to them (P12, Phase 3)

Listening to a person with dementia and their carer seemed to promote person‐centred learning, prompting participants to *see the person* rather than focusing on their dementia.

(A) person is more than their dementia…Dementia does not define the person (P5, Phase 1)

*New Ways of Seeing* needed conditions to be established. These conditions were represented by the following sub categories:

- Adaptive Thinking
- Building Relationships
- Transformative Learning

These sub categories were defined and confirmed over time, and although they are presented as discreet categories, they are all interrelated as illustrated in Figure 2.

### 3.4 Adaptive thinking

Growing awareness of observing the abilities of the person with dementia rather than their disabilities, appeared to challenge participants' preconceptions and stereotypes resulting in focusing on the person. Experiences seemed to enhance personal and professional growth of participants by challenging previous beliefs of dementia. Participants seemed motivated to reflect on their practice and their development as adult nurses, which resulted in changes to their approaches in their practice:

I've seen other people in practice and I don't always agree with how they treat people with dementia…they get cross, but shouting at people and getting cross doesn't do anything does it? There's been a couple of times I have stepped in – I am like, I'll deal with this person. I feel confident…not to hold back. (P3, Phase 3)

Participants made frequent references throughout the study to *focusing less on the task* and *doing*. Instead they spoke of planning and providing care that enabled and affirmed the person, all of which is in line with Kitwood's concept of person‐centred care.^28^

Dementia in hospital, focus is on doing. Care is very task driven, and you don't know anything about them, you just see the patient in front of you…at home you see the person (P11, Phase 2)

Participants demonstrated perspective‐taking throughout their visits, discussing how they could imagine themselves in *their shoes*. Instead of viewing people with dementia as a homogenous group, participants began to see the person as an individual rather than just their dementia.

### 3.5 Building relationships

It could be argued that building relationships is one of the most important skills that students learn, participants shared examples of how their experiences enabled them to specifically adapt to the needs of the person with dementia, playing to their individual strengths, and being proactive in their approaches to make a difference. Findings suggest that new insights into dementia, led participants to focus less on their *drive to do* (tasks), to recognise the importance of *being with* the person (*presencing*) and *building relationships* **.** Visits were a powerful catalyst for developing authentic connections that lay the foundation for person‐centred care. As Beckett et al.^25^ (2007) proposes; *Nurses make a difference as much by the ways they relate interpersonally to patients as they do by the technical interventions they use*.

Biggest impact for me has been the realisation…sometime people do not want answers, they just want people who will listen… realisation of how important human interaction is…importance of building relationships (P1, Phase 2).

Participants reflected on the importance of *being with* rather than *doing to,* which Fazio et al.^28^ advocates is fundamental to person‐centred care. The resulting impact was seen in changes to participants' practice; illustrated by their change of focus from *working on tasks* to *working with*, from *managing* to *actively listening to patients*.

### 3.6 Transformative learning

Visits with people with dementia and their carers enhanced students' understanding and perceptions of the experiences of dementia and led to new insights and attitudinal changes. As a result of their new insights into dementia, participants recognised the importance of focusing less on their drive to do (*tasks*), and their focus changed to *being present*. The impact of visits resulted in transformative learning; demonstrated by the participants' self‐examination of their practice and examples shared of their changes to practice.

I was like comparing myself between the visits and then in practice…I am much more confident in practice now… I am not afraid to try different approaches…knowing that we can make a difference. (P5, Phase 3)

Participant 8 shared a story of how she felt that she had made a real difference during her visits. Because of concerns expressed by the carer about the residential home his wife was attending, she suggested and assisted in putting together a ‘This is Me’ book to help staff. Based on his concerns, they included information such as what his wife likes to drink and eat, what activities she enjoys doing along with a summary of her life history. On a subsequent visit, her husband shared how much of a difference he felt that the book had made to support her care and how he did not worry when she went for respite, for the first time he felt could relax and start to enjoy activities that he used to do:

He said it's (*book*) is helping her…when she goes to respite home and she can just point at the picture if she can't remember…made me feel happy as he seemed much happier about her going to care, it felt like we had made a difference.[…] made me appreciate the impact of the way we care can have. (P8, Phase 2)

Findings suggest that listening to the individual stories of people with dementia and their carers as well as observing the impact dementia had on their lives stimulated learning affectively, experientially and cognitively. Participant's experiences appeared transformative as their perceptions of dementia were challenged and changed:

There should be less taboo about dementia. Programmes such as Time for Dementia are transformative, gives the opportunity to slowly face our misconceptions … gives the new generation of healthcare professionals a better understanding of how to go about approaching dementia as a whole. (P12, Phase 3)

Hearing the lived experience of dementia moved academic learning away from traditional lectures into the real‐life domain and learning from ‘*real life’* was easier to remember.

Lectures have their place, I mean it's good for when you're learning about the digestive system and all that, but, when you're learning about experiences… it was invaluable to go to someone's own home, to hear and to actually see that from their perspective. (P6, Phase 3)

## 4 DISCUSSION

The skills' gap in undergraduate nurse education in dementia care is an international issue^29^ ^,^ ^12^ ^,^ ^14^ ^,^ ^30^ ^,^ ^31^ The findings of this study suggest that one way to address this issue is by the inclusion of programmes such as *Time for Dementia* in undergraduate healthcare curricula. This contributes to the body of knowledge of dementia education that working in partnership and involving people with dementia in healthcare education can be transformative in creating a positive dementia discourse and promote positive practice.^27^

The development of the theory of *Whole Sight* as a representation of nursing students *New Ways of Seeing* dementia and their role in supporting people with dementia and their carers is of potential use in understanding how *Time for Dementia* may exert a positive effect. *New Ways of Seeing* dementia, led to personal and professional growth demonstrated by their adaptive thinking, as assumptions of dementia were challenged. *Whole Sight* opens new directions for future research into how to improve dementia education, offering novel perspectives that can be taken into consideration in developing dementia education in healthcare curricula. The results of this study suggest that visits created a positive dementia discourse, shifting the focus from seeing dementia as a disability to a more balanced view of dementia, whereby participants could see potential and hope. This contrasts with Webber and Robinson^31^ literature review which evaluated the effectiveness of service user involvement in social work education. They found that although 29 of the papers reviewed reported evidence that students valued the involvement of service users none showed evidence of changes in student's practice. Service users were involved in classroom‐based sessions and Webber and Robinson^31^ concluded that empowerment of service users was the prominent outcome rather than any value to student's education experience. This suggests that it is not just involvement but the nature of service user involvement that is of importance to changing practice.

For many, the impact of the experience of getting to know the person with dementia in their own homes over 3 years was transformational. Participants were able to develop an understanding of the importance of *being* (engaging with the person) rather than just *doing (*performing tasks) in practice which contributed to how they saw themselves as future nurses.

## 5 LIMITATIONS

This study was limited to students of adult nursing participating in *Time for Dementia* in one Health Education Institute. This and the small number of participants involved means that there are limitations to the generalisation of the findings directly to a wider population of healthcare professionals in training. However, this was a qualitative study and the depth of the interviews and their being repeated gives support to the value of the framework of understanding the changes made by *Time for Dementia* to the students and the theory generated. A strength of this study is that it used an inductive rather than deductive approach to data collection and analysis. This made the experiences of student participants' central to the development of a theory for understanding the impact of involving people with dementia and their carers in education. A further limitation is that students were a group willing to participate in the research. However, they made their choice at the beginning of the programme and the interviews covered their experiences prospectively, so we did not only sample those with a positive experience. A final limitation is that although people with dementia helped to inform this study through their involvement with the wider steering group they had no other involvement in this study.

## 6 CONCLUSIONS

Dementia is a global challenge and how well we respond to the projected doubling of the numbers of people with dementia in the next generation will depend to a large extent on the quality of our future healthcare workforce. People with dementia are commonly seen only as recipients and not active participants in care and education. Here we explored the impact of a novel approach to change this, the *Time for Dementia* Programme. As a result of visits, student nurse participants reframed their perceptions of dementia. Participants recognised the person with dementia as separate to, and more than their presenting illness. This increasing awareness led to a person‐centred shift in practice, demonstrated by examples of a more humanistic approach to supporting people with dementia in their clinical practice. This study offers new insights in how we might develop dementia education that focuses on interconnectedness and caring relationships, promoting a *Whole Sight* focus on the person rather than on their dementia.

Incorporating community based educational initiatives like *Time for Dementia* into healthcare education may help build a workforce that meets the needs of people with dementia and their carers. The lessons learnt in dementia may well generalise to people with other long‐term conditions and the frailty and multimorbidity often found in later life. Further research is needed to explore whether the intervention has the same effect in other student healthcare professionals, on attitudes to other long‐term conditions, and on its effect in practice once students qualify.

## CONFLICT OF INTEREST

None declared.

## DATA AVAILABILITY

The data that support the findings of this study are openly available University of Surrey Research Insight: https://epubs.surrey.ac.uk/851797/, reference number: http://doi.org/10.15126/thesis.00851797.
